# Detection of occludable angle with anterior segment optical coherence tomography and Pentacam as non-contact screening methods

**DOI:** 10.1007/s10792-021-02208-y

**Published:** 2022-01-19

**Authors:** Javier Benitez-del-Castillo, Ali Nowrouzi, Mario Rodriguez-Calzadilla, Inmaculada Mota-Chozas, Maria Dolores Pinazo-Duran

**Affiliations:** 1 Department of Ophthalmology, Hospital Universitario de Jerez de la Frontera, 11407 Jerez de la Frontera, Spain; 2 Researchers Red Temática de Investigación Cooperativa en Patología Ocular (OFTARED) RD16/0008/0022, Instituto de Salud Carlos III, 28029 Madrid, Spain; 3 Ophthalmic Research Unit Santiago Grisolia/FISABIO, 46017 Valencia, Spain

**Keywords:** Occludable angle, AS-OCT, Pentacam

## Abstract

**Purpose:**

To evaluate diagnostic capacity for occludable anterior chamber angle detection with anterior segment optical coherence tomography (AS-OCT) and Pentacam.

**Methods:**

Observational cross-sectional study with AS-OCT and Pentacam. AS-OCT measures: angle opening distance from Schwalbe line (SL) perpendicular (AOD-SL-Perp) and vertical to iris (AOD-SL-Vert), and iridotrabecular angle (ITA). Pentacam measures: anterior chamber depth (ACD), anterior chamber volume (ACV), and anterior chamber angle (ACA). We analysed Spearman’s correlation with gonioscopic classification. Area under receiver operating characteristic curves (AUCs) for occludable angle detection were compared. Agreement between iridocorneal values of methods was evaluated.

**Results:**

Seventy-four left eyes of 74 patients. Correlation between temporal AS-OCT and gonioscopy: 0.83 (*p* < 0.0001) AOD-SL-Perp temporal, 0.82 (*p* < 0.0001) AOD-SL-Vert temporal, and 0.69 (*p* < 0.0001) ITA temporal. Correlation between AS-OCT nasal and gonioscopy: 0.74 (*p* < 0.0001) AOD-SL-Perp nasal, 0.74 (*p* < 0.0001) AOD-SL-Vert nasal, and 0.70 (*p* < 0.0001) ITA nasal. Correlation of Pentacam with temporal gonioscopy: 0.57 (*p* < 0.0001) ACD, 0.56 (*p* < 0.0001) ACV, and 0.63 (*p* < 0.0001) ACA. Correlation of Pentacam with nasal gonioscopy: 0.47 (IC 0.27–0.73, *p* < 0.0001) ACD, 0.49 (*p* < 0.0001) ACV, and 0.56 (CI 0.38–0.7, *p* < 0.0001) ACA. AS-OCT AUCs: AOD-SL-Perp temporal 0.89 (CI 0.80–0.95), AOD-SL-Vert 0.87 (CI 0.77–0.94), ITA temporal 0.88 (CI 0.78–0.94), AOD-SL-Perp nasal 0.83 (CI 0.72–0.91), AOD-SL-Vert nasal 0.87 (CI 0.77–0.94), and ITA nasal 0.91 (IC 0.81–0.96). Pentacam AUCs: ACD 0.76 (CI 0.64–0.85), ACV 0.75 (CI 0.63–0.84), and ACA 0.84 (CI 0.74–0.92). No statistical differences between different AUCs. Intraclass correlation coefficient (ICC) of ACA (Pentacam) with ITA temporal (AS-OCT) 0.59 and with nasal ITA nasal (AS-OCT) 0.65.

**Conclusion:**

Both systems show high capacity for non-contact occludable angle detection. But agreement between methods is moderate or low.

## Introduction

Glaucoma is the leading cause of irreversible blindness worldwide [1–3]. Primary open-angle glaucoma (POAG) predominates over primary angle-closure glaucoma (PACG) in most population-based studies: prevalence of POAG is calculated to be 3.54% in those between 40 and 80 years old [4], and prevalence of PACG is approximately 0.92% [2]. In contrast to the higher prevalence of POAG, PACG has a threefold greater risk of developing blindness compared to POAG [4–9]: people bilaterally blind from glaucoma are increasing worldwide, and it has been estimated to be 5.9 million due to POAG and 5.3 million due to PACG in 2020 [2].

Prevalence of PACG varies across geographic regions and ethnic groups [10, 11], and consistently with previous studies [2], the prevalence of PACG is highest in Asia (1.09%) [4]. According to other systematic reviews, prevalence of PACG in people over 40 years old, from European-derived populations, is estimated in 0.4% around year 2012 [12].

As damage by acute angle closure is irreversible and can be severe, screening for occludable angles is important. Furthermore, it is admitted that proper laser peripheral iridotomy (LPI) prevents occludable angles from the angle closure attack, eliminating the relative pupillary block component, and should be recommended as a prophylactic treatment for all occludable angles [13].

Gonioscopy is the gold standard for identifying occludable angles. It takes a certain exploration time and manipulation of the eye with a gonioscopy lens and the consequent risk of infection [14]. It is also a relatively subjective technique: findings may vary with the amount of light or mechanical compression used during eye examination and require the expertise of a trained examiner. Intra- and interobserver reproducibility is poor [5, 6].

We currently have non-contact imaging devices capable to explore the ocular anterior segment, providing a rapid visualization and measurements of the anterior chamber angle (ACA): the anterior segment optical coherence tomography (AS-OCT) device and the anterior segment camera based on the Scheimpflug technology device (Pentacam). There are many papers in the scientific literature that study this particular clinical application, but few have recently studied and compared these method’s diagnostic ability [15–26]. Therefore, the objective of this study is to evaluate the diagnostic capacity for occludable anterior chamber angle detection of AS-OCT and Pentacam as non-contact screening tests in our population.

## Methods

### Subjects and measurement protocol

Patients who met the eligibility criteria were consecutively enrolled in this observational cross-sectional study performed at the Ophthalmology Department of the Hospital Universitario del S.A.S. de Jerez (Cádiz, Spain). The study protocol was approved by the ethics and clinical research committee (CEI/HUJ001/2020) which was in agreement with the revised provisions of the Declaration of Helsinki. Written informed consent was provided to patients.

We included patients referred for the first time to the Glaucoma Unit, between 20 and 80 years old, with or without ocular hypotensive medical treatment and classified as open- or closed-angle glaucoma or suspects. We excluded patients with clinical manifestations of closed-angle glaucoma, previous intraocular surgeries or traumas, corneal or anterior segment abnormalities, and those who previously underwent laser iridotomies.

All patients underwent a complete ophthalmic examination, including slit-lamp biomicroscopy and gonioscopic evaluation. Anterior segment was also studied with 3D Maestro-1 OCT TOPCON (Topcon, Tokyo, Japan) and Pentacam Scheimpflug images (Pentacam, Oculus Inc., Wetzlar, Germany). Anterior segment images were obtained before performing the gonioscopy examination. We randomly selected the left eye of each participant for all measurements.

All gonioscopy examinations were performed by an experienced glaucoma specialist (JBC) using a classic 1-mirror (62°) Goldmann goniolens. The examination was performed in a dark room (room light off), with the lowest intensity beam on a Haag Streit (Koeniz, Switzerland) BM 900 slit lamp with a width of 0.4 to 0.5 mm and length of 8 mm, as usual in practice. Grading was recorded for the 4 angle quadrants (superior, inferior, nasal, and temporal) in both eyes of each subject, also as usual, using the modified Shaffer grade which was based on the original Shaffer system [27] as described by other authors [24, 25]: Grade 4, wide open angle (around 40 degrees) with a flat or concave iris surface and the scleral spur (SS) visible without directing the eye toward the gonioscopy mirror; Grade 3, wide open angle (30 degrees) with a slightly convex iris surface and the SS visible without directing the eye toward the gonioscopy mirror; Grade 2, open angle (20 degrees) with a convex iris surface and the SS visible without directing the eye toward the gonioscopy mirror; Grade 1, narrow angle (10 degrees) with a convex iris surface and the SS visible only redirecting the eye toward the gonioscopy mirror; Grade 0.5, slit angle (less than 10 degrees) and the SS not visible even redirecting the eye toward the gonioscopy mirror; and Grade 0, closed angle and the SS not visible even redirecting the eye toward the gonioscopy mirror. An occludable angle was defined as modified Shaffer Grade ≤ 1 in two or more than two quadrants.

The 3D Maestro-1 OCT from Topcon uses an advanced automated optical coherence tomography system and a fundus camera for assessing ocular pathologies. The full-colour fundus camera provides both 2D and 3D pictures in high resolution, and the Spectral Domain OCT offers 50,000 A-scans each second. A forehead separator is mounted for anterior segment imaging. All scans were performed under uniform conditions of dim illumination between 8 and 10 LUX (standardized using a light meter app for phone: Lux Light Meter®, Doggo Apps, Moscow, Russia). The patient was guided to look at the centre (no external light fixation is needed), and then by manual mode of AS-OCT, both nasal and temporal extreme were captured. Angle images were captured using the horizontal linear scan protocol (from 3-o'clock to 9-o'clock direction) because it can be taken more easily than those of superior and inferior quadrants and do not need eyelid manipulation [28]. All measurements were repeated at least twice until sufficient quality is obtained. This first set of images for each eye were acquired and examined by the same investigator (AN). The location of SL was manually identified by the shadow of the anterior extreme of trabecular meshwork (TM) by the termination of the corneal endothelium. Images in which Schwalbe line (SL) landmark was not detectable by two experienced ophthalmologists (AN, JBC) were finally excluded. To determine interobserver reproducibility, angle measurements were independently taken on the images obtained in this initial examination by the second experienced observer (JBC) and to determine intraobserver reproducibility the first observer (AN) repeated the scanning with OCT and the angle measurements obtaining a second set of images using the same protocol one week after the first examination.

**Fig. 1:**
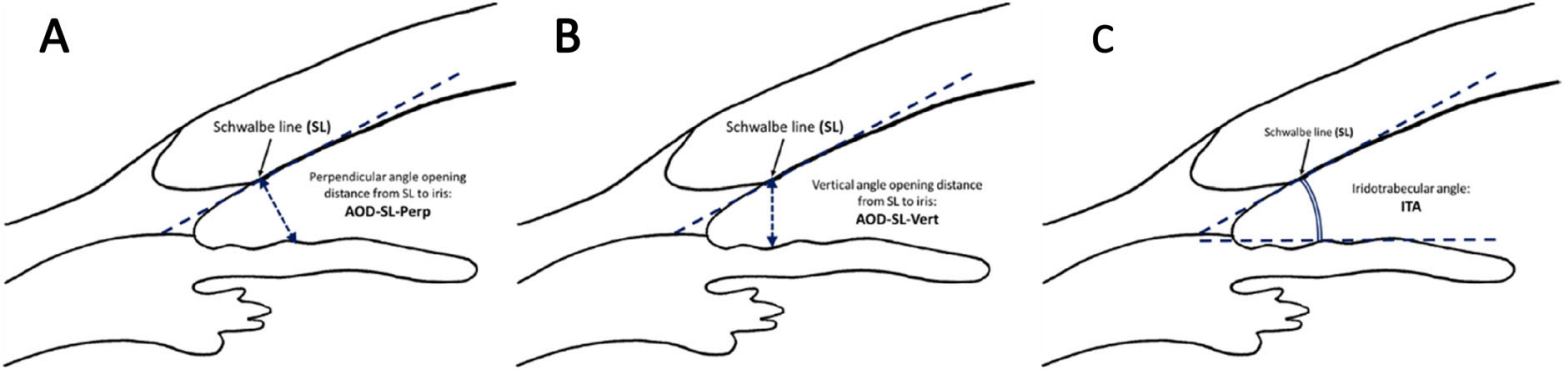
Anterior segment optical coherence tomography AS-OCT parameters measured. **a**: perpendicular angle opening distance from Schwalbe line (SL) to iris (AOD-SL-Perp). **b**: vertical angle opening distance from Schwalbe line (SL) to iris (AOD-SL-Vert). **c**: iridotrabecular angle (ITA). ( Modified from Cheung et al. 24)

Measurements collected with AS-OCT were: the perpendicular angle opening distance from SL (AOD-SL-Perp), the distance measured perpendicularly to a line drawn from the SS to the SL, from SL to the iris surface, in the temporal and nasal side, measured in microns (Fig. 1a); the vertical angle opening distance from SL (AOD-SL-Vert), the distance measured vertical to iris surface from SL, in the temporal and the nasal side, also in microns (Fig. 1b); and the iridotrabecular angle (ITA) in degrees, both temporal and in the nasal side (Fig. 1c). The AOD-SLs were measured by computer callipers. TIA was measured manually with math angle protractor between iris surface tangent line and a line drawn from SS to SL. The AOD-SL-Perp and AOD-SL-Vert distances were only measured in images in which SL was decided as detectable by both observers (AN and JBC), as mentioned before.

Pentacam is a topograph with a rotational Scheimpflug camera that captures 50 images in less than 2 s. It produces high-resolution tridimensional images of the anterior segment of the eye. Anterior segment examination with Pentacam is performed in the same measured light conditions. The variables collected are automatically recorded by the device: the anterior chamber depth (ACD), measured from the corneal endothelium (interior ACD), in mm; the anterior chamber volume (ACV), measured from endothelium to the iris and crystalline surface, in mm^3^ and the anterior chamber angle (ACA) which is the smallest of the two camera angles, temporal and nasal quadrants, measured in the horizontal axis (180°), in degrees. A second examination was performed if measurements could not be obtained.

### Statistical analysis

Sample size calculation was based on the assumption that a value of 0.83 of AUC (area under the ROC curves) is statistically significant to detect occludable angles (mean value of different AS-OCT and Pentacam parameters published [29]), with a null hypothesis value of 0.5. Given a type I error of 0.05 and a type II error of 0.20, 67 patients were required as the total sample size. A minimum recruitment of 70 patients was planned to allow for possible patient exclusions.

Measurements of AS-OCT and Pentacam and other continuous variables are described as mean values and standard deviations (SD). The Kolmogorov–Smirnov test was used to determine the normal distribution of the variables measured. Univariate correlations between AS-OCT and Pentacam measured parameters and gonioscopic classification (Shaffer classification) were established by Spearman’s Rho test.

The intraclass correlation coefficient (ICC) was calculated to evaluate both inter- and intraobserver reproducibility for AS-OCT parameters.

Receiver operating characteristic (ROC) curves were constructed for each AS-OCT and Pentacam parameter for detecting occludable angles, cut-off values, sensitivity, specificity that were calculated, and area under the ROC curves (AUCs) were used to compare the discriminating ability between AS-OCT and Pentacam. The method of DeLong [30] was used to compare the AUC of the different study parameters.

Agreement between iridocorneal values in degrees of different systems has been evaluated also with intraclass correlation coefficient (ICC), and Bland–Altman plots were constructed.

Significance was set at *p* < 0.05.

Statistical tests were performed using the software package MedCalc v19.1 (MedCalc Software, Mariakerke, Belgium).

## Results

Seventy-four left eyes of seventy-four different patients were enrolled in the study (46 females, 62.1%, and 28 males, 27.8%, *P* = 0.09) with a mean age of 62,6 years (SD = 8.4). In total, 57 (77%) eyes were classified as open-angle eyes and 17 (22.9%) as occludable angle eyes (Table 1). Finally, 70 patients (94.5%) for temporal values and 68 patients (91.8%) for nasal values were eligible (positive identification SL with AS-OCT). All of them showed also recognizable measurements with Pentacam.

**Table 1:** Clinical characteristics of the study population (mean and standard deviation). Anterior segment optical coherence tomography (AS-OCT) parameters: perpendicular angle opening distance from Schwalbe line (SL) to iris (AOD-SL-Perp temporal and nasal), vertical angle opening distance from Schwalbe line (SL) to iris (AOD-SL-Vert temporal and nasal), and iridotrabecular angle (ITA temporal and nasal). Pentacam anterior segment parameters: anterior chamber depth (ACD), anterior chamber volume (ACV), and anterior chamber angle (ACA)

	All eyes (*n* = 74)	Open-angle eyes (*n* = 57)	Occludable angle eyes (*n* = 17)	Significance level P
AGE (years) (SD)	62.60 (8.44)	62.73 (8.22)	62.17 (9.37)	*P* = 0.8120
SEX				*P* = 0.0947
Male	28	25	3	
Female	46	32	14	
AS-OCT measurements	All eyes (*n* = 74)	Open-angle eyes (*n* = 57)	Occludable angle eyes (*n* = 17)	Significance level P
AOD-SL-Perp temporal (microns) (SD)	352.67 (161.14)	391.92 (157.79)	208.73 (60,79)	*P* < 0.0001
AOD-SL-Perp nasal (microns) (SD)	351.75 (150.48)	386.64 (148.51)	228.46 (74.52)	*P* = 0.0002
AOD-SL-Vert temporal (microns) (SD)	324.91 (128.34)	355.16 (127.04)	214 (46.41)	*P* = 0.0001
AOD-SL-Vert nasal (microns) (SD)	306.13 (120.85)	336.20 (116.52)	199.86 (62.77)	*P* < 0.0001
ITA temporal (degrees) (SD)	26.01 (8,86)	28.49 (8,33)	17.6 (4.35)	*P* < 0.0001
ITA nasal (degrees) (SD)	25.24 (7.55)	27.62 (6.8)	17.13 (2.85)	*P* < 0.0001
Pentacam measurements	Al eyes (*n* = 74)	Open-angle eyes (*n* = 57)	Occludable angle eyes (*n* = 17)	Significance level P
ACD (mm) (SD)	2.59 (0.46)	2.67 (0.38)	2.32 (0.61)	*P* = 0.0070
ACV (mm3) (SD)	130.62 (39.65)	139.09 (36.12)	104.23 (39.5)	*P* = 0.0012
ACA (degrees) (SD)	30.3 (6.2)	31.97 (5.66)	25.1 (4.87)	*P* < 0.0001

All values obtained with AS-OCT showed clear statistical differences between eyes classified as open-angle eyes and occludable angle eyes (Table 1).

All segment anterior parameters obtained with Pentacam also showed clear statistical differences between eyes classified as open-angle eyes and occludable angle eyes (Table 1).

ICCs values for evaluating intraobserver reproducibility of AS-OCT parameters range from 0.979 of ITA nasal to 0.998 of AOD-SL-Perp temporal. ICCs values for evaluating interobserver reproducibility of AS-OCT parameters range from 0.968 of ITA nasal to 0.983 of AOD-SL-Perp nasal (Table 2).

**Table 2:** Upper part: intra- and interobserver reproducibility of AS-OCT parameters (intraclass correlation coefficient, ICC, and 95% confidence interval). Lower part: Spearman’s correlation between temporal and nasal anterior segment optical coherence tomography (AS-OCT) values and gonioscopy and correlation between Pentacam values and temporal and nasal gonioscopy. AS-OCT parameters: perpendicular angle opening distance from Schwalbe line (SL) to iris (AOD-SL-Perp temporal and nasal), vertical angle opening distance from Schwalbe line (SL) to iris (AOD-SL-Vert temporal and nasal), and iridotrabecular angle (ITA temporal and nasal). Pentacam anterior segment parameters: anterior chamber depth (ACD), anterior chamber volume (ACV), and anterior chamber angle (ACA)

Intraclass correlation coefficient	Intraobserver (95% CI)	Interobserver (95% CI)
AOD-SL-Perp temporal	0.9999 (0.9998 to 0.9999)	0.9794 (0.9671 to 0.9872)
AOD-SL-Perp nasal	0.9899 (0.9837 to 0.9938)	0.9837 (0.9737 to 0.9899)
AOD-SL-Vert temporal	0.9983 (0.9972 to 0.9989)	0.9749 (0.9599 to 0.9843)
AOD-SL-Vert nasal	0.9898 (0.9835 to 0.9937)	0.9682 (0.9489 to 0.9802)
ITA temporal	0.9835 (0.9732 to 0.9899)	0.9764 (0.9617 to 0.9855)
ITA nasal	0.9796 (0.9668 to 0.9874)	0.9682 (0.9486 to 0.9804)
Correlation AS-OCT with temporal Gonioscopy	Correlation Coefficient (95% CI)	Significance Level P
AOD-SL-Perp temporal	0.831	< 0.0001
AOD-SL-Vert temporal	0.821	< 0.0001
ITA temporal	0.693	< 0.0001
Correlation AS-OCT with nasal gonioscopy	Correlation Coefficient (95% CI)	Significance Level P
AOD-SL-Perp nasal	0.745	< 0.0001
AOD-SL-Vert nasal	0.746	< 0.0001
ITA nasal	0.707	< 0.0001
Correlation Pentacam with temporal gonioscopy	Correlation Coefficient (95% CI)	Significance Level P
ACD	0.571	< 0.0001
ACV	0.564	< 0.0001
ACA	0.632	< 0.0001
Correlation Pentacam with nasal gonioscopy	Correlation Coefficient (95% CI)	Significance Level P
ACD	0.475	< 0.0001
ACV	0.494	< 0.0001
ACA	0.569	< 0.0001

Correlation between temporal AS-OCT values and gonioscopy was 0.83 (*P* < 0.0001) for AOD-SL-Perp temporal, 0.82 (*P* < 0.0001) for AOD-SL-Vert temporal, and 0.69 (*P* < 0.0001) for ITA temporal (Table 2). Correlation between AS-OCT nasal values and gonioscopy was 0.74 (*P* < 0.0001) for AOD-SL-Perp nasal, 0.74 (*P* < 0.0001) for AOD-SL-Vert nasal, and 0.70 (*P* < 0.0001) for ITA nasal (Table 2).

Correlation of Pentacam values with temporal gonioscopy were 0.57 (*P* < 0.0001) for ACD, 0.56 (*P* < 0.0001) for ACV, and 0.63 (*P* < 0.0001) for ACA (Table 2). Correlation of Pentacam values with nasal gonioscopy was 0.47 (*P* < 0.0001) for ACD, 0.49 (*P* < 0.0001) for ACV, and 0.56 (*P* < 0.0001) for ACA (Table 2).

**Fig. 2:**
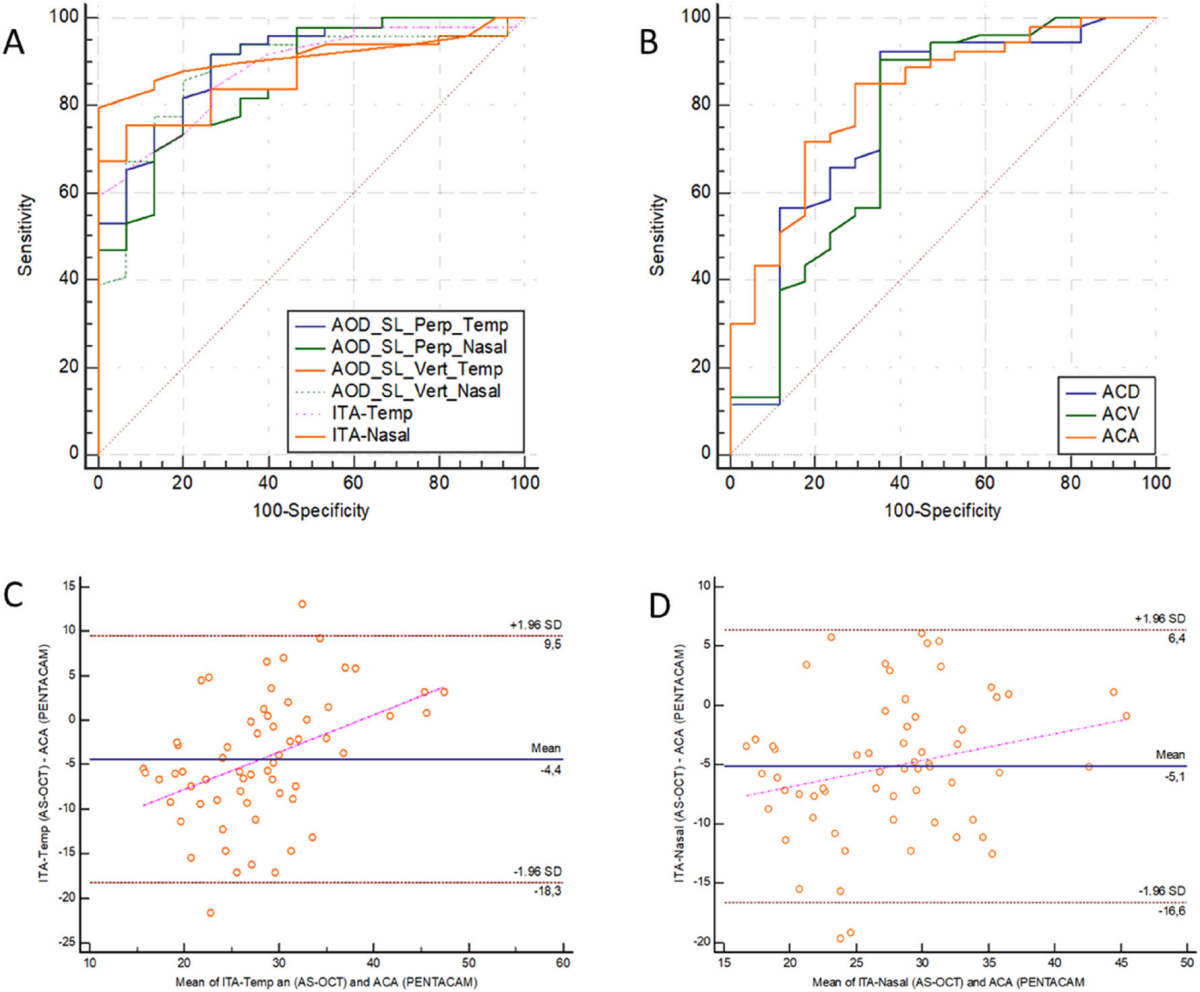
**a**: Receiver operating curves (ROC) of different anterior segment optical coherence tomography (AS-OCT) parameters. **b**: Receiver operating curves (ROC) of different Pentacam parameters. **c**: Bland–Altman plots showing the agreement between the trabecular-iris angle (ITA) measurements by Pentacam and by AS-OCT in the temporal quadrant (regression equation: *y* = −16.2835 + 0.4229 x; coefficient of determination *R*^2^: 0.174; *p* = 0.0007). D: Bland–Altman plots showing the agreement between the trabecular-iris angle (ITA) measurements by Pentacam and by AS-OCT in the nasal quadrant (regression equation: *y* = −11.3866 + 0.2259 x; coefficient of determination R^2^: 0.062; *p* = 0.05). AS-OCT parameters: perpendicular angle opening distance from Schwalbe line (SL) to iris (AOD-SL-Perp temporal and nasal), vertical angle opening distance from Schwalbe line (SL) to iris (AOD-SL-Vert temporal and nasal), and iridotrabecular angle (ITA temporal and nasal). Pentacam anterior segment parameters: anterior chamber depth (ACD), anterior chamber volume (ACV), and anterior chamber angle (ACA)

We have obtained the following AUCs values for occludable angle detection with AS-OCT: AOD-SL-Perp temporal 0.89, AOD-SL-Vert 0.87, ITA temporal 0.88, AOD-SL-Perp nasal 0.83, AOD-SL-Vert nasal 0.87, and ITA nasal 0.91. There is not a statistical difference among them (Table 3 and Fig. 2a).

**Table 3:** Diagnostic capability of different anterior segment optical coherence tomography (AS-OCT) and Pentacam parameters. AUC: area under the ROC curve (receiver operating curves) and 95% confidence interval. Cut-off values, sensitivity (%), and specificity (%). AS-OCT parameters: perpendicular angle opening distance from Schwalbe line (SL) to iris (AOD-SL-Perp temporal and nasal), vertical angle opening distance from Schwalbe line (SL) to iris (AOD-SL-Vert temporal and nasal), and iridotrabecular angle (ITA temporal and nasal). Pentacam anterior segment parameters: anterior chamber depth (ACD), anterior chamber volume (ACV), and anterior chamber angle (ACA)

Diagnostic capability AS-OCT:	AUCs	95% CI AUC	Cut-off value	Sensitivity (%)	Specificity (%)
AOD-SL-Perp temporal (microns)	0.898	0.802 to 0.957	230	90.91	73.33
AOD-SL-Perp nasal (microns)	0.834	0.724 to 0.913	194	90.11	53.33
AOD-SL-Vert temporal (microns)	0.874	0.773 to 0.941	273	76.36	93.33
AOD-SL-Vert nasal (microns)	0.876	0.774 to 0.944	205	92.45	73.33
ITA temporal (degrees)	0.886	0.783 to 0.951	26	60.78	100
ITA nasal (degrees)	0.913	0.818 to 0.968	23	78.43	100
Diagnostic capability Pentacam	AUCs	95% CI AUC	Cut-off value	Sensitivity (%)	Specificity (%)
ACD (mm)	0.764	0.648 to 0.857	2.16	92.45	64.71
ACV (mm^3^)	0.754	0.636 to 0.849	97	90.57	64.71
ACA (degrees)	0.822	0.712 to 0.903	26.5	84.91	70.59

We have obtained the following AUCs values for occludable angle detection with Pentacam: ACD 0.76, ACV 0.75, and ACA 0.84. There is not a statistical difference among them (Table 3 and Fig. 2b).

There are no statistical differences between different AUROC values obtained with the two different methods: AS-OCT and Pentacam (Table 4).

**Table 4:** Pairwise comparison of receiver operating curves (ROC) of different anterior segment optical coherence tomography (AS-OCT) and Pentacam parameters. AS-OCT parameters: perpendicular angle opening distance from Schwalbe line (SL) to iris (AOD-SL-Perp temporal and nasal), vertical angle opening distance from Schwalbe line (SL) to iris (AOD-SL-Vert temporal and nasal), and iridotrabecular angle (ITA temporal and nasal). Pentacam anterior segment parameters: anterior chamber depth (ACD), anterior chamber volume (ACV), and anterior chamber angle (ACA)

Pairwise comparison of ROC curves		AOD-SL-Perp temporal	AOD-SL-Vert temporal	ITA temporal	AOD-SL-Perp nasal	AOD-SL-Vert nasal	ITA nasal	ACD Pentacam	ACV Pentacam	ACA Pentacam
AOD-SL-Perp temporal	Dif. between areas Std Error 95% CI Sig level		0.03 0.03 − 0.04 to 0.1 P = 0.4053	0.01 0.05 − 0.01 to 0.12 P = 0.7767	0.04 0.03 − 0.02 to 0.11 P = 0.21	0.02 0.03 − 0.04 to 0.08 P = 0.5023	0.01 0.05 − 0.09 to 0.11 P = 0.8486	0.05 0.06 − 0.07 to 0.18 P = 0.4226	0.07 0.07 − 0.06 to 0.22 P = 0.2844	0.10 0.07 − 0.04 to 0.26 P = 0.1762
AOD-SL-Vert temporal	Dif. between areas Std Error 95% CI Sig level	0.03 0.03 0,04 to 0.1 P = 0.4053		0.01 0.05 − 0.08 to 0.11 P = 0.7824	0.01 0.04 − 0.07 to 0.10 P = 0.8037	0.01 0.03 − 0.06 to 0.08 P = 0.7659	0.04 0.04 − 0.03 to 0.12 P = 0.2778	0.02 0.06 − 0.10 to 0.15 P = 0.7460	0.04 0.07 − 0.10 to 0.20 P = 0.5402	0.07 0.07 − 0.06 to 0.21 P = 0.3105
ITA temporal	Dif. between areas Std Error 95% CI Sig level	0.01 0.05 − 0.01 to 0.12 P = 0.7767	0.01 0.05 − 0.08 to 0.11 P = 0.7824		0.02 0.06 − 0.10 to 0.15 P = 0.6992	0.00 0.06 − 0.11 to 0.12 P = 0.9733	0.02 0.02 − 0.02 to 0.08 P = 0.5516	0.03 0.06 − 0.08 to 0.15 P = 0.6012	0.05 0.07 − 0.08 to 0.20 P = 0.4141	0.08 0.06 − 0.04 to 0.22 P = 0.2094
AOD-SL-Perp nasal	Dif. between areas Std Error 95% CI Sig level	0.04 0.03 − 0.02 to 0.11 P = 0.21	0.01 0,04 − 0.07 to 0.10 P = 0.8037	0.02 0.06 − 0.10 to 0,15 P = 0.6992		0.02 0.03 − 0.05 to 0.09 P = 0.5516	0.05 0.05 − 0.06 to 0.17 P = 0.3489	0.01 0,06 − 0.12 to 0.14 P = 0.8544	0.04 0.07 − 0.10 to 0.18 P = 0.5882	0.06 0.08 − 0.10 to 0.23 P = 0.4324
AOD-SL-Vert nasal	Dif. between areas Std Error 95% CI Sig level	0.02 0.03 − 0.04 to 0.08 P = 0.5023	0.01 0.03 − 0.06 to 0.08 P = 0.7659	0.00 0.06 − 0.11 to 0.12 P = 0.9733	0.02 0.03 − 0.05 to 0.09 P = 0.5516		0,03 0,05 − 0,06 to 0,13 P = 0,5252	0,02 0,05 − 0,08 to 0,13 P = 0,6744	0,05 0,06 − 0,07 to 0,18 P = 0,4309	0,08 0,07 − 0,06 to 0,22 P = 0,2939
ITA nasal	Dif. between areas Std Error 95% CI Sig level	0.01 0.05 − 0.09 to 0.11 P = 0.8486	0.04 0.04 − 0.03 to 0.12 P = 0.2778	0.02 0.02 − 0.02 to 0.08 P = 0.5516	0.05 0.05 − 0.06 to 0.17 P = 0.3489	0.03 0.05 − 0.06 to 0.13 P = 0.5252		0.05 0.05 − 0.05 to 0.16 P = 0.3566	0.07 0.06 − 0.05 to 0.21 P = 0.2555	0.10 0.06 − 0.02 to 0.23 P = 0.1089
ACD Pentacam	Dif. between areas Std Error 95% CI Sig level	0.05 0.06 − 0.07 to 0.18 P = 0.4226	0.02 0.06 − 0.10 to 0.15 P = 0.7460	0.03 0.06 − 0.08 to 0.15 P = 0.6012	0.01 0.06 − 0.12 to 0.14 P = 0.8544	0.02 0.05 − 0.08 to 0.13 P = 0.6744	0.05 0.05 − 0.05 to 0.16 P = 0.3566		0.01 0.02 − 0.04 to 0.06 P = 0.7	0.05 0.09 − 0.13 to 0.25 P = 0.5579
ACV Pentacam	Dif. between areas Std Error 95% CI Sig level	0.07 0.07 − 0.06 to 0.22 P = 0.2844	0.04 0.07 − 0.10 to 0.20 P = 0.5402	0.05 0.07 − 0.08 to 0.20 P = 0.4141	0.04 0.07 − 0.10 to 0.18 P = 0.5882	0.05 0.06 − 0.07 to 0.18 P = 0.4309	0.07 0.06 − 0.05 to 0.21 P = 0.2555	0.01 0.02 − 0.04 to 0.06 P = 0.7		0.06 0.09 − 0.11 to 0.24 P = 0.4588
ACA Pentacam	Dif. between areas Std Error 95% CI Sig level	0.10 0.07 − 0.04 to 0.26 P = 0.1762	0.07 0.07 − 0.06 to 0.21 P = 0.3105	0.08 0.06 − 0.04 to 0.22 P = 0.2094	0.06 0.08 − 0.10 to 0.23 P = 0.4324	0.08 0.07 − 0.06 to 0.22 P = 0.2939	0.10 0.06 − 0.02 to 0.23 P = 0.1089	0.05 0.09 − 0.13 to 0.25 P = 0.5579	0.06 0.09 − 0.11 to 0.24 P = 0.4588	

Agreement between iridocorneal temporal and nasal measured with AS-OCT and Pentacam was analysed with ICC of ACA of Pentacam with temporal ITA of AS-OCT which was 0.59 (95% CI 0.4–0.79) and with nasal ITA of AS-OCT which was 0.65 (95% CI 0.49–0.77). Agreement between AS-OCT and Pentacam was also studied by drawing individual Bland–Altman plots (Fig. 2c and d). Plots show only a moderate or low agreement between OCT and Pentacam ITA measurements, with a mean difference of -4.4 degrees for the temporal quadrant and −5.1 degrees for the nasal quadrant (*P* < 0.001). Figure 2c shows the difference plot for ACA of Pentacam with temporal ITA of AS-OCT, and Fig. 2d shows the difference plot for ACA of Pentacam with nasal ITA of AS-OCT. As it is shown in the graphics, agreement level between methods is moderate and low, respectively, because of a proportional error that increases as the mean values of angle degrees increase.

## Conclusions

To our knowledge, this is the first study that analyses the diagnostic ability of AS-OCT SL-based parameters and Pentacam anterior segment measurements for the detection of gonioscopic occludable angles in a Caucasian population. We have found that both non-contact imaging systems have a high capacity for this purpose.

Anterior chamber angle evaluation is of great importance because glaucoma prevalence and morbidity can be expected to increase with the world's population ageing and we recognize PACG as a severe but potentially preventable disease [2, 4, 5]. But, as mentioned, gonioscopy has some limitations, e.g. manipulation of the eye is required with the consequent safety risk. Using non-contact imaging methods has advantages for the patient and the examiner, particularly in this current SARS-Cov2 pandemic.

We have used a Fourier domain-OCT (FD-OCT) to measure angle parameters referenced to the SL [24, 25]. Other studies, including those in a recent systematic review [26] and those combining AS-OCT and Pentacam [21–23, 29], used classic SS-based AS-OCT parameters. But some studies have reported that SS cannot be identified in 20%–25% of the time domain-OCT images [11, 31]. FD-OCT uses 830 nm with some less tissue penetration, but higher resolution and SL could be more reliable than the SS in assessing the angle. Furthermore, measuring the AOD from SL could more directly reflect the accessibility of the trabecular meshwork (TM) to aqueous humour compared with estimations based on a fixed distance from the SS [32].

In this study, SL was visible in 94.5% of temporal images and 91.8% of nasal images, a similar rate of SL visibility reported in the literature of 95% [24] and 97% [25], and also a similar percentage of usable images to measure iridocorneal angle SL-based parameters referred in a large-scale review of technical artefacts in AS-OCT [33].

Demographic characteristics of our population, the used AS-OCT device, and the parameters measured in the study could limit the comparison of our results with other similar studies which include Asian origin patients [23–26, 29] and/or analyse SS-based AS-OCT parameters [21–23, 29].

Mean values of AC angle in degrees in our study with AS-OCT, ITA temporal 26 ± 8.8 degrees and ITA nasal 25.2 ± 7.5 degrees, and with Pentacam, ACA 30.3 ± 6.2 degrees, are lower than those of a similar study in a Caucasian population only, ITA temporal 35.8 ± 13.2 degrees and ITA nasal 35.7 ± 12.9 degrees with AS-OCT, and ACA temporal 35.7 ± 7.3 degrees and ACA nasal 36.4 ± 8.2 degrees with Pentacam [21, 22]. Although the percentage of women is similar in both populations (62%), in our study we not included patients previously treated with LPI and mean age of our population is more than a decade older, 49.1 ± 15.2 versus 62.6 ± 8.4 years old [34]. Anyway, there are no statistical differences in age (*P* = 0.81) and sex distribution (*P* = 0.09) between open-angle eyes and occludable angle eyes in our population.

All AS-OCT and Pentacam values obtained show clear statistical differences between eyes classified as open-angle eyes and occludable angle eyes (Table 1).

Although it has been recently published that consistent and reproducible ACA measurements could be obtained from multiple AS-OCT devices including both FD-OCT and TD-OCT [35], we consider that not requiring an external fixation light for the examination, as with the 3D Maestro-1 OCT from Topcon, may contribute to the high reproducibility of measurements we have found [36].

Correlation between AS-OCT values and gonioscopy grade is high and ranges from 0.70 to 0.83 and similar to other authors analysing SL-based parameters with AS-OCT [24, 25]. Correlation between Pentacam values and gonioscopy grade is also rather high and ranges from 0.47 to 0.63, higher to another study that found only moderate associations in an Asian Indian population [29], but similar to correlation values achieved in a study in Caucasian population [22].

AUCs of AS-OCT parameters for diagnosing occludable angles are high ranging from 0.83 to 0.91, similar to other authors found analysing SL-based parameters with AS-OCT [24, 25]. AUCs of Pentacam parameters for diagnosing occludable angles are also ranging from 0.75 to 0.82, similar to other authors that analysed different populations [23, 29]. There are no statistical differences between different AUCs values obtained with the two methods, AS-OCT and Pentacam, showing a similar high capacity for occludable angle detection based on the AUCs values found in the study (Tables 3 and 4).

Agreement found between iridocorneal angle measurements of AS-OCT and Pentacam (degrees) is only moderate and low, as other authors previously reported [21, 37]. Values obtained with these two different methods are not interchangeable: Bland–Altman figures show that the Pentacam tends to overestimate the measurements of narrow angles and underestimate that of open angles in comparison with OCT measurements as others found [21]. Possible explanations for these findings are on the one hand, the different nature of the two methods in the acquisition and interpretation of the images, and on the other hand, although the AS-OCT and Pentacam examinations are performed under the same light conditions, the intimate intensity of light during each examination must inevitably be different from one to another, as Pentacam uses visible light to image the angle [38]. It has been clearly shown varying intensity of light can open the angle to a different degree, especially in patients with an occludable angle [39–42].

Our study has several limitations. The study group is not population-based and is limited to Caucasian patients. Sample size may be too small for certain sub-analyses such as correlations with other factors or definitive conclusions on specific cut-off points values for the measured parameters. Regarding the obtained images we have to take into account that they are captured only in the horizontal cross section and could not be representative of other quadrants. Angle landmarks have to be manually identified in the images, and this could introduce some subjective variability in the measured parameters. Finally, these images are static anterior ocular segment biometric measurements that allow us a quantitative, but not qualitative evaluation and, of course, do not reflect the dynamic nature of the anterior segment angle.

An important practical issue to consider is the high cost of these technologies, which are not accessible in all countries around the world. The results of our study are obviously not applicable in these regions, despite being hit by the pandemic like the others. To date, there are no cost–benefit studies in this regard.

In conclusion, AS-OCT iridocorneal angle parameters, also SL-based measurements, and Pentacam iridocorneal parameters measured have shown a high capacity for gonioscopic occludable angle detection in practice as non-contact screening methods. However, to date, none of the used imaging methods provides sufficient information about the anterior chamber angle anatomy to be considered as a complete substitute for gonioscopy [26, 43]. Improvement in image analysis algorithms, that could be based in artificial intelligence [44], or even new generation of imaging devices to explore, not only morphology but also structural mechanics about angle closure, are still unmet needs in ophthalmology. It will notably improve the efficiency and accuracy of angle examinations in clinical studies.
